# Tobacco Price Increase and Smoking Cessation in Japan, a Developed Country With Affordable Tobacco: A National Population-Based Observational Study

**DOI:** 10.2188/jea.JE20140183

**Published:** 2016-01-05

**Authors:** Takahiro Tabuchi, Masakazu Nakamura, Tomio Nakayama, Isao Miyashiro, Jun-ichiro Mori, Hideaki Tsukuma

**Affiliations:** 1 Center for Cancer Control and Statistics, Osaka Medical Center for Cancer and Cardiovascular Diseases, Osaka, Japan; 2 Department of Health Promotion and Prevention, Osaka Center for Cancer and Cardiovascular Disease Prevention, Osaka, Japan; 3 Center for Medical Education, Shinshu University School of Medicine, Matsumoto, Nagano, Japan

**Keywords:** tobacco price increase, smoking cessation, Japan, linkage study

## Abstract

**Background:**

Longitudinal assessment of the impact of tobacco price on smoking cessation is scarce. Our objective was to investigate the effect of a price increase in October 2010 on cessation rates according to gender, age, socioeconomic status, and level of tobacco dependence in Japan.

**Methods:**

We used longitudinal data linkage of two nationally representative studies and followed 2702 smokers for assessment of their cessation status. The odds ratios (ORs) for cessation were calculated using logistic regression. To estimate the impact of the 2010 tobacco price increase on cessation, data from 2007 were used as a reference category.

**Results:**

Overall cessation rates significantly increased from 2007 to 2010, from 3.7% to 10.7% for men and from 9.9% to 16.3% for women. Cessation rates were 9.3% for men who smoked 1–10 cigarettes per day, 2.7% for men who smoked 11–20 cigarettes per day, and 2.0% for men who smoked more than 20 cigarettes per day in 2007. These rates increased to 15.5%, 10.0%, and 8.0%, respectively, in 2010. The impact was stronger among subjects who smoked more than 11 cigarettes per day than those who smoked 1–10 cigarettes per day in both sexes: ORs for 2010 were 4.04 for those smoking 11–20 cigarettes per day, 4.26 for those smoking more than 20 cigarettes per day, and 1.80 for those smoking 1–10 cigarettes per day in the main model in men. There were no obvious differences in the relationship between tobacco price increase and smoking cessation across age and household expenditure groups.

**Conclusions:**

The tobacco price increase in Japan had a significant impact on smoking cessation in both sexes, especially among heavy smokers, with no clear difference in effect by socio-demographic status.

## INTRODUCTION

Assessment of the impact of tobacco price increases on changes in smoking behaviors in different social groups is a priority in health policy research.^1^^,^^2^ Tobacco taxation (generally accompanying a price increase for tobacco products) has been considered the best practice for reducing population tobacco use and inherent smoking inequality.^3^^,^^4^ From a population health perspective,^5^ a population-based intervention, such as a countrywide tobacco price increase, is expected to affect not the only the affluent population but also high-risk and vulnerable populations. Previous studies have shown that tobacco price increases reduce tobacco use and smoking inequality because they have a stronger influence on the poor and the young than the affluent and the old in developed countries, such as the United States, Scotland, and Australia.^4^ However, mixed results were also reported for differences in age, gender, and education.^2^^,^^6^ Moreover, a high rate of tobacco dependence has been shown to strongly predict low rates of smoking behavior change, including smoking cessation.^7^

Since the tobacco tax was established in Japan in 1998, the tobacco tax/price has been increased three times (July 1, 2003; July 1, 2006; and October 1, 2010). Therefore, the price of a pack of 20 of the most popular brand of cigarette in Japan, Mild Seven, increased from 250 yen to 270 yen (8% increase) in 2003, to 300 yen (11% increase) in 2006, and to 410 yen (37% increase) in 2010.^8^ The tobacco industry has also increased the price for its own benefit. Partly as a result of these price increases, adult smoking prevalence in Japan has declined: the proportion of current smokers has decreased from 48% in 2001 to 33% in 2010 among men, and from 14% in 2001 to 10% in 2010 among women.^9^ However, according to the affordability index, the price of tobacco was considered to be very low in Japan in 2009.^10^ Of all developed countries surveyed, cigarettes were most affordable in Japan in 2009 (people only had to work for 11.5 minutes to earn the price of a pack of 20 cigarettes).^10^ Even after the 2010 price increase, this figure was expected to be around 16 minutes, whereas in other developed countries, such as Australia, Canada, and the Netherlands, it was 30 minutes in 2009.^10^ Because the affordability of tobacco in Japan may create special conditions for smokers, the impact of a tobacco price increase on smoking behavior and smoking inequality should be specifically assessed. Very few longitudinal studies have examined the effect of tobacco price on rates of smoking cessation,^4^^,^^11^^,^^12^ and the present paper is the first to use linkage data (ie, used as longitudinal data) to explore this effect in Japan.

Our objective was to investigate the impact of tobacco price increases on rates of smoking cessation according to and adjusted for different variables, such as gender, age, socioeconomic status and level of tobacco dependence, in Japan, a developed country with affordable tobacco.^10^

## METHODS

### Data

We used linkage data from the 2007 and 2010 versions of the Comprehensive Survey of Living Conditions of People on Health and Welfare (CSLC) and the National Health and Nutritional Survey (NHNS), which were conducted by the Japanese Ministry of Health, Labour and Welfare (MHLW). The CSLC collects information on health-related factors, such as smoking behaviors, every 3 years in the first week of June, while the NHNS collects information on smoking behaviors annually in November (on a weekday). Out of 940 000 inhabited census tracts (the sampling unit for national census in 2005), 5440 were randomly sampled across Japan in 2007 (5510 in 2010, independently from 2007) for the collection of CSLC data from all household members within each census tract. Of the tracts selected for CLSC, 300 were randomly selected for the NHNS; therefore, some CLSC respondents were interviewed again 5 months later in the NHNS (Figure).

**Figure.**
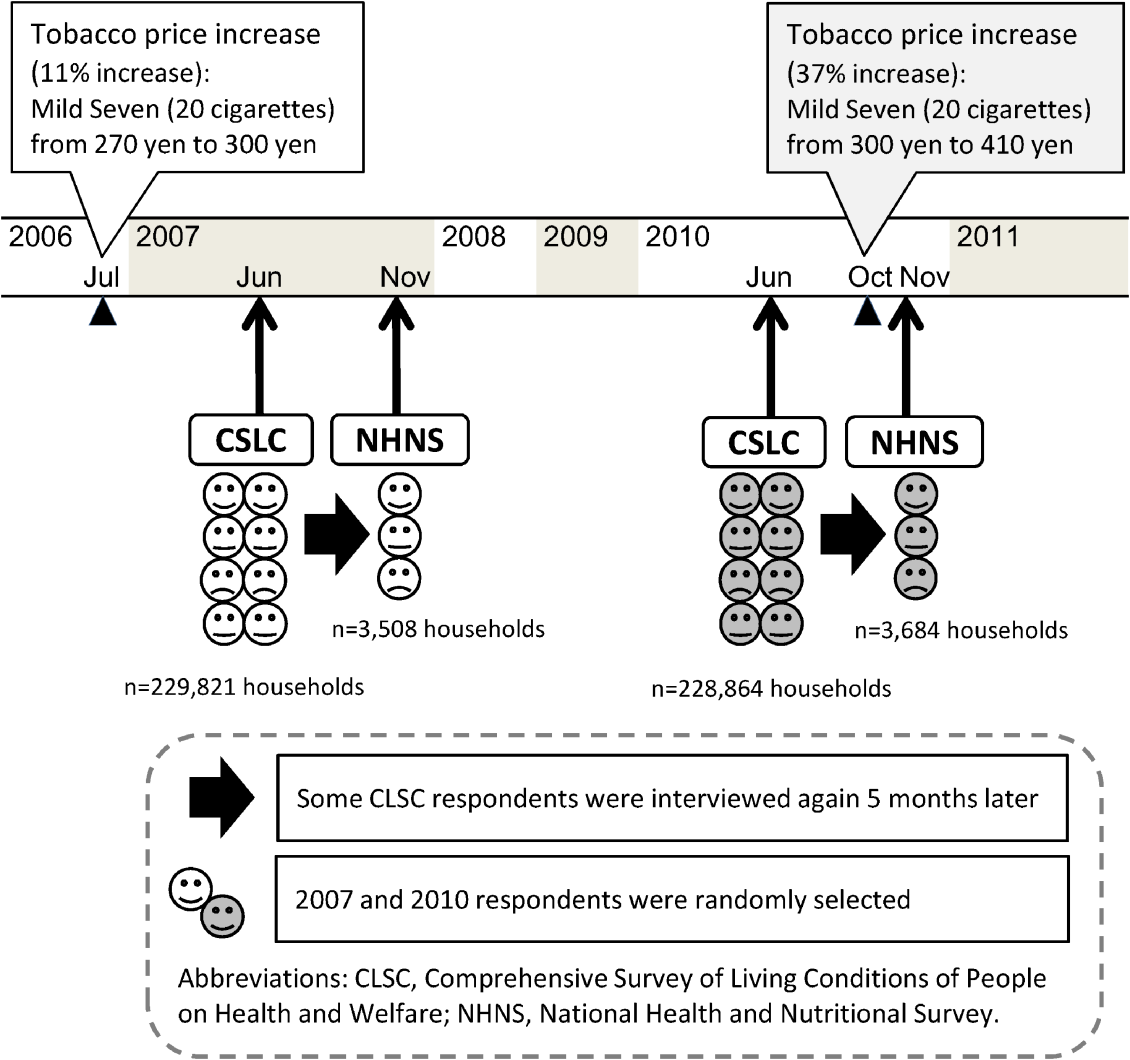
Time frame around the study.

Data were available for 229 821 (response rate: 79.9%) households in 2007 and 228 864 (79.1%) households in 2010 for the CSLC^9^ and 3508 (66.5%) households in 2007 and 3684 (68.8%) in 2010 for the NHNS.^13^^,^^14^ Linkage data within each year from subjects who responded to both surveys with smoking information and who were aged 20–79 years at baseline were analyzed. Of 11 088 non-institutionalized eligible subjects (2639 men and 2995 women in 2007; 2598 men and 2856 women in 2010), 2702 were current smokers at baseline in the present study (1080 men and 355 women in 2007 and 961 men and 306 women in 2010). Data were used with permission from MHLW. Analyses of national survey data are exempt from ethical review according to the Epidemiological Research Guidelines.

### Smoking

First, individuals from the 2007 and 2010 CSLC were categorized as current smokers or non-current smokers at baseline. In the CSLC, smoking habits were assessed based on the following four categories: (a) “I don’t smoke”; (b) “I smoke every day”; (c) “I smoke occasionally but not every day”; and (d) “I have stopped smoking for more than 1 month”. We categorized (b) and (c) as current smokers (as of June). The NHNS divided subjects into “ever smokers” and “never smokers” and asked, “Do you now smoke cigarettes every day, some days, or not at all (stopped smoking more than 1 month)?” We categorized ever smokers who reported smoking “every day” or “some days” as current smokers (as of November).

Current smoker prevalence was defined as the rate of current smokers among study subjects (considering June or November timing of outcome assessment as subgroups). Smoking cessation was identified by smokers in June (CSLC) who were no longer smokers in November (NHNS). The smoking cessation rate was calculated as the percentage of current smokers (as of June) who did not smoke at the time of the NHNS (as of November): that is, the percentage of “non-current smokers” in the NHNS among current smokers in the CSLC.

### Variables

Variables related to smoking behaviors (identified from the CSLC) were used to identify the characteristics of the baseline study subjects and to control for their possible confounding effects (if they met the requirements for confounding). In line with previous studies,^15^^,^^16^ we used age, household expenditure, housing tenure (home-owner or not), employment status (working or not), marital status (married, never married, or widowed/divorced), self-rated health (poor or not), number of cigarettes smoked per day (1–10, 11–20, or >20), and number of other household smokers (0 or ≥1) (see supplementary methods in eMaterial). The number of cigarettes smoked per day was regarded as a proxy indicator for tobacco dependence.^17^^,^^18^ The cutoffs of 10 and 20 cigarettes were used because the CSLC questionnaire had categories of 1–10, 11–20, 21–30, and ≥31 cigarettes, and we modified the category on the basis of its distribution.

### Statistical analysis

As the tobacco price increase occurred during the period between the CSLC (June) and the NHNS (November) in 2010, we had a unique opportunity to analyze the effect of the increase. Statistical analyses were conducted separately for men and women, because there are large gender differences in smoking behaviors in most Asian countries, including Japan.^19^ Basic characteristics and smoking cessation rates were tabulated according to the above-mentioned variables. Fisher’s exact test was used to compare the differences in subject characteristics and cessation rates between 2007 and 2010.

Univariate logistic regression among a pooled sample of participants in 2007 and 2010 (separately for men and women) was applied to calculate crude odds ratios (ORs) with 95% confidence intervals (CIs) for smoking cessation. Multivariate logistic regression was used to calculate adjusted ORs. To estimate the impact of the October 2010 tobacco price increase on smoking cessation, cessation status in 2007 was used as a reference category (ie, 2007 versus 2010), because there was no price increase during the 5 months of follow-up from June to November in 2007.

In addition to sex separation, stratified analyses were implemented to estimate whether the association between tobacco price increase and smoking cessation rates varied according to age, socioeconomic status, or tobacco dependence. We used household expenditure as a socioeconomic variable in the stratified analysis, because expenditure is an analog of income (ie, a representative socioeconomic factor) in Japan.^20^ Subjects with a missing value for cigarettes smoked per day were excluded from the regression analyses, while a missing value category on expenditure was used as a dummy variable because of the high frequency of missing expenditure values. In addition, to confirm the interaction effect between tobacco price and personal factors, such as age, we also conducted multivariate analyses using interaction terms. We modified the interaction term according to the results of the stratified analyses, generating a new dichotomized category; for example, cigarettes smoked per day of 11 or more (ie, both “11–20” and “>20”) were scored as 1 for the “2010/Cigarettes per day of 11 or more” category and as 0 for the remaining three combination categories (eg, “2007/Cigarettes per day of 1–10”).

Probability values for statistical tests were two tailed, and *P* < 0.05 was regarded as statistically significant. All statistical analyses were performed using SAS version 9.2 (SAS Institute, Cary, NC, USA).

## RESULTS

The prevalence of current smoking in June 2007 and 2010 among all subjects (including smokers and non-smokers) is shown in eTable 1. The overall prevalence of current smoking in June significantly decreased from 2007 to 2010 for men (40.9% to 37.0%) but not for women, although prevalence decreased by approximately 10% in both sexes from 2007 to 2010.

Basic characteristics of current smokers in June, comparing 2007 with 2010, are shown in Table 1. A statistically significant difference in the distribution between 2007 and 2010 was observed only in “cigarettes smoked per day” in men. All other variables were not significantly different between 2007 and 2010. Based on these results, we treated “cigarettes smoked per day” as a confounding factor for men in subsequent multivariate analyses.

**Table 1.** Basic characteristics of current smokers in June, comparison between 2007 and 2010 (baseline)

Characteristics	Men			Women		
	
	2007 *n* = 1080	2010 *n* = 961	*P* for difference^a^	2007 *n* = 355	2010 *n* = 306	*P* for difference^a^
Cigarettes per day			0.009			0.257
1–10	205 (19.0)	219 (22.8)		121 (34.1)	125 (40.9)	
11–20	523 (48.4)	490 (51.0)		186 (52.4)	150 (49.0)	
>20	348 (32.2)	249 (25.9)		45 (12.7)	29 (9.5)	
Missing	4 (0.4)	3 (0.3)		3 (0.9)	2 (0.7)	
Other household smoker(s)			0.509			0.813
None	733 (67.9)	639 (66.5)		147 (41.4)	130 (42.5)	
One or more	347 (32.1)	322 (33.5)		208 (58.6)	176 (57.5)	
Household expenditure			0.369			0.434
1st (lowest) tertile	335 (31.0)	293 (30.5)		115 (32.4)	98 (32.0)	
2nd tertile	338 (31.3)	316 (32.9)		104 (29.3)	99 (32.4)	
3rd (highest) tertile	338 (31.3)	307 (32.0)		108 (30.4)	94 (30.7)	
Missing	69 (6.4)	45 (4.7)		28 (7.9)	15 (4.9)	
Age group, years			0.230			0.727
20–39	378 (35.0)	317 (33.0)		149 (42.0)	122 (39.9)	
40–59	459 (42.5)	397 (41.3)		151 (42.5)	130 (42.5)	
60–79	243 (22.5)	247 (25.7)		55 (15.5)	54 (17.7)	
Home owner			0.165			0.678
No	226 (20.9)	226 (23.5)		118 (33.2)	97 (31.7)	
Yes	854 (79.1)	735 (76.5)		237 (66.8)	209 (68.3)	
Employment status			0.621			0.662
Working	926 (85.7)	811 (84.4)		239 (67.3)	197 (64.4)	
Not working	142 (13.2)	136 (14.2)		113 (31.8)	107 (35.0)	
Missing	12 (1.1)	14 (1.5)		3 (0.9)	2 (0.7)	
Marital status			0.174			0.967
Married	834 (77.2)	710 (73.9)		226 (63.7)	198 (64.7)	
Never married	191 (17.7)	201 (20.9)		69 (19.4)	57 (18.6)	
Widowed/Divorced	55 (5.1)	50 (5.2)		60 (16.9)	51 (16.7)	
Poor self-rated health			0.354			0.466
No	914 (84.6)	826 (86.0)		281 (79.2)	254 (83.0)	
Yes	129 (11.9)	97 (10.1)		62 (17.5)	44 (14.4)	
Missing	37 (3.4)	38 (4.0)		12 (3.4)	8 (2.6)	

^a^*P* for difference was calculated by Fisher’s exact test.

Rates of smoking cessation among smokers after 5 months of follow-up, according to basic characteristics, are shown in Table 2. Overall cessation rates significantly differed between 2007 and 2010 (3.7% to 10.7% for men and 9.9% to 16.3% for women). Smoking cessation rates were 9.3% for men who smoked 1–10 cigarettes per day, 2.7% for men who smoked 11–20 cigarettes per day, and 2.0% for men who smoked more than 20 cigarettes per day in 2007; these rates increased to 15.5%, 10.0%, and 8.0%, respectively, in 2010. A statistically significant difference in cessation rates between 2007 and 2010 was observed in the following groups: those who smoked 11–20 cigarettes per day, the highest household expenditure tertile, non-home-owners, and married participants of both sexes; and men who smoked more than 20 cigarettes per day, did not live with other household smokers, who lived with one or more household smokers, all age groups, home-owners, who were working, and who did not have poor self-rated health.

**Table 2.** Smoking cessation rates (during 5 months of follow-up) and increase (absolute value) in 2010 compared with 2007 among smokers according to baseline characteristics

Characteristics	Smoking cessation rates (%) among smokers							

	Men				Women			
	
	2007 %	2010 %	Increase % point	*P* for difference^a^	2007 %	2010 %	Increase % point	*P* for difference^a^
Overall population	3.7	10.7	7.0	<0.001	9.9	16.3	6.5	0.014
Cigarettes per day								
1–10	9.3	15.5	6.3	0.057	18.2	22.4	4.2	0.432
11–20	2.7	10.0	7.3	<0.001	5.9	13.3	7.4	0.023
>20	2.0	8.0	6.0	0.001	2.2	3.5	1.2	Not applicable
Other household smoker(s)								
None	4.1	11.3	7.2	<0.001	12.2	18.5	6.2	0.180
One or more	2.9	9.6	6.8	0.000	8.2	14.8	6.6	0.051
Household expenditure								
1st (lowest) tertile	3.0	12.0	9.0	<0.001	11.3	12.2	0.9	0.835
2nd tertile	3.0	8.5	5.6	0.002	5.8	13.1	7.4	0.092
3rd (highest) tertile	4.7	12.4	7.7	0.001	10.2	24.5	14.3	0.008
Age group, years								
20–39	3.7	10.1	6.4	0.001	9.4	16.4	7.0	0.098
40–59	2.8	9.8	7.0	<0.001	8.6	13.9	5.2	0.184
60–79	5.4	13.0	7.6	0.005	14.6	22.2	7.7	0.332
Home owner								
No	2.7	9.7	7.1	0.003	5.9	14.4	8.5	0.041
Yes	4.0	11.0	7.0	<0.001	11.8	17.2	5.4	0.107
Employment status								
Working	3.1	10.7	7.6	<0.001	8.4	13.2	4.8	0.118
Not working	7.0	11.0	4.0	0.297	13.3	22.4	9.2	0.081
Marital status								
Married	3.1	11.6	8.4	<0.001	11.1	19.7	8.6	0.015
Never married	5.8	8.5	2.7	0.332	8.7	14.0	5.3	0.401
Widowed/Divorced	5.5	8.0	2.6	0.706	6.7	5.9	−0.8	Not applicable
Poor self-rated health								
No	3.7	10.4	6.7	<0.001	11.0	16.9	5.9	0.080
Yes	3.1	9.3	6.2	0.060	4.8	13.6	8.8	0.158

^a^*P* for difference was calculated by Fisher’s exact test.

Note: Some increases do not match the results using these figures due to rounding up.

Table 3 shows logistic regression results for 2010 compared with 2007 for smoking cessation rates according to baseline stratification. The cigarettes smoked per day-adjusted model (ie, the crude model in the cigarettes smoked per day stratification) was considered an appropriate model (main model) to account for confounding in men, while the unadjusted crude model was used in women. For men, significant ORs for cessation for the period of June to November 2010 were observed in all stratifications except for 1–10 cigarettes smoked per day and did not change materially after additional adjustment for age group (adjustments for other variables also did not significantly change the results; data not shown). The ORs for cessation were stronger among men than among women (ORs of 3.01 [95% CI, 2.06–4.39] for men and 1.80 [95% CI, 1.13–2.87] for women in the main model). The ORs for cessation were higher among subjects of both sexes who smoked 11–20 or more than 20 cigarettes per day than among those smoked 1–10 cigarettes per day (eg, ORs of 4.04 [95% CI, 2.20–7.42] for 11–20 cigarettes smoked per day, 4.26 [95% CI, 1.77–10.23] for more than 20 cigarettes smoked per day, and 1.80 [95% CI, 0.99–3.27] for 1–10 cigarettes smoked per day in the main model in men). There were no obvious differences in the relationships between the stratified gradation of household expenditure tertiles or age groups and the impact of the tobacco price increase on smoking cessation for either sex (eg, the OR for cessation was highest in the lowest expenditure tertile than in the highest expenditure tertile in men, while the inverse was true in women). In additional models using an interaction term, the interaction term “2010/Cigarettes smoked per day of 11 or more” category showed a significant OR of 2.32 (95% CI, 1.06–5.05) in men. No other interaction terms were significant in either sex (data not shown).

**Table 3.** Crude and adjusted odds ratios for year 2010 (versus 2007) for smoking cessation among smokers

Stratification variable	OR (95% CI)	

	Men	Women
	2010 (ref = 2007)	2010 (ref = 2007)
***Total***	*n* = 2034	*n* = 656
Crude	3.12 (2.14, 4.55)	1.80 (1.13, 2.87)^a^
Cigarettes per day-adjusted	3.01 (2.06, 4.39)^a^	1.68 (1.04, 2.70)
Age and cigarettes per day-adjusted	3.09 (2.12, 4.50)	1.78 (1.12, 2.85)

***1–10 cigarettes per day***	*n* = 424	*n* = 246
Crude	1.80 (0.99, 3.27)^a^	1.30 (0.70, 2.43)^a^
Age-adjusted	1.84 (1.01, 3.35)	1.29 (0.69, 2.41)
***11–20 cigarettes per day***	*n* = 1013	*n* = 336
Crude	4.04 (2.20, 7.42)^a^	2.45 (1.13, 5.29)^a^
Age-adjusted	3.93 (2.14, 7.23)	2.40 (1.11, 5.19)
***>20 cigarettes per day***	*n* = 597	*n* = 74
Crude	4.26 (1.77, 10.23)^a^	1.57 (0.09, 26.15)^a^
Age-adjusted	4.15 (1.72, 10.00)	NC

***Household expenditure, lowest***	*n* = 628	*n* = 212
Crude	4.41 (2.14, 9.07)	1.00 (0.43, 2.35)^a^
Cigarettes per day-adjusted	4.52 (2.19, 9.35)^a^	NC
Age and cigarettes per day-adjusted	4.50 (2.18, 9.31)	NC
***Household expenditure, middle***	*n* = 650	*n* = 201
Crude	3.07 (1.46, 6.45)	2.47 (0.90, 6.79)^a^
Cigarettes per day-adjusted	2.92 (1.38, 6.17)^a^	2.82 (1.00, 7.94)
Age and cigarettes per day-adjusted	2.94 (1.39, 6.22)	2.84 (1.01, 8.03)
***Household expenditure, highest***	*n* = 642	*n* = 201
Crude	2.84 (1.55, 5.20)	2.83 (1.29, 6.18)^a^
Cigarettes per day-adjusted	2.69 (1.45, 4.99)^a^	NC
Age and cigarettes per day-adjusted	2.67 (1.43, 4.97)	NC

***Age group, 20–39 years***	*n* = 691	*n* = 267
Crude	2.94 (1.54, 5.62)	1.94 (0.92, 4.11)^a^
Cigarettes per day-adjusted	2.64 (1.37, 5.08)^a^	NC
***Age group, 40–59 years***	*n* = 856	*n* = 280
Crude	3.74 (1.97, 7.11)	1.69 (0.80, 3.61)^a^
Cigarettes per day-adjusted	3.55 (1.86, 6.76)^a^	NC
***Age group, 60–79 years***	*n* = 487	*n* = 109
Crude	2.60 (1.33, 5.08)	1.68 (0.63, 4.50)^a^
Cigarettes per day-adjusted	2.77 (1.41, 5.47)^a^	1.65 (0.60, 4.51)

CI, confidence interval; NC, not converged; OR, odds ratio; ref, reference category.

^a^Models that were considered as main models accounting for confounding.

## DISCUSSION

The tobacco price increase in Japan, which was implemented in October 2010, was found to be associated with significantly increased cessation rates in 2010 compared with rates in 2007. Our findings support the notion that tobacco price increase is one of the best practices for advancing tobacco control.^2^ The tobacco price increase was estimated to increase absolute smoking cessation rates by 7.0% for men and 6.5% for women during 5 months in 2010 compared with the same time period in 2007. Since there are no local-level price variations and the regulated elevated tobacco price was applied concurrently at the time of tobacco taxation and industrial price increase within Japan under the Tobacco Business Law^21^ (ie, with no time-delay of the market price increase overall Japan), all subjects were assumed to be affected by the price increase in 2010. Although the tax was increase on October 1, 2010, at least 1 month before the survey in November 2010, the intervention may have had an impact prior to its implementation in practice (eg, via anticipation effects).^22^ In fact, according to a survey of cessation intention among smokers in August–September 2010 (ie, immediately before the tax increase),^23^ 53% of smokers intended to quit because of the tax increase (1 or 2 months after the survey), and 72% of cessation-intending smokers reported that they intended to quit by the day of the tax increase. Therefore, cessation during June to November in 2010 was evaluated as a total effect of the tobacco price increase compared with 2007. Because there were no major smoking restrictions or other tobacco control measures between 2007 and 2010 (except medication for nicotine dependence using varenicline and nicotine patches, which started to be sold in 2008) in Japan,^21^ we considered 2007 to be a reasonable reference category.

The ORs for smoking cessation after implementation of the price increase were higher among men than among women. Although women showed higher absolute cessation rates than men in both 2007 and 2010, tobacco price increase may have a more powerful effect on smoking cessation in men than in women. This is possibly because men have relatively less disposable money than women,^24^ women are more health conscious than men,^25^ and women may have more occasions to stop smoking, such as during a pregnancy, than men.^26^ Because no other major tobacco control policies were implemented between 2007 and 2010 in Japan,^21^ the price increase appears likely to have been the cause of the increased cessation rates.

Generally, tobacco dependence inhibits smoking cessation.^7^ However, the effect of tobacco dependence on the association between tobacco price increase and smoking cessation was previously unknown.^4^ In the current study, the price increase showed higher ORs for smoking cessation among those who smoked more heavily, although we did not focus on women who smoke more than 20 cigarettes per day, because they showed wide variance due to the small number of outcomes (*n* = 2) in the category. The additional analyses using interaction terms confirmed our interpretation of the results using stratification analyses, although the non-significance of the interaction terms in women did not mean that there was no interaction; this lack of an interaction effect might be due in part to the small sample size. When the number of cigarettes smoked per day was interpreted as a proxy of tobacco dependence, the analysis yielded a surprising tendency toward cessation among heavier smokers, in contrast to the expectation that tobacco dependence inhibits smoking cessation.^7^^,^^12^ This result may be because the increase in tobacco price has a greater impact on those who smoke more heavily. Further, smokers who were likely to be sensitive to tobacco price increases and who were likely to have stopped smoking might have continued to smoke, as tobacco remains affordable in Japan despite the price increases.^10^

### Policy implication

The Health Japan 21 (second version), a health promotion strategy in Japan, prioritized the reduction of smoking prevalence and health inequality, including smoking inequality.^27^ The strategy’s primary target for smoking prevalence was 12% among adults by 2022. In 2010, NHNS showed a large reduction in smoking prevalence in Japan due to the 2010 tobacco price increase, but the 2011 NHNS (which enrolled an independent sample from the 2010 survey) reported an increased smoking prevalence compared with 2010 (from 19.5% to 20.1% for adults of both sexes).^14^ Among smokers who quit, only a small proportion succeed long-term^28^; this relapse might be due in part to the low tobacco price in Japan, even after the price increase in 2010. A previous study in California revealed that the impact of a price increase only lasted for 4 months after the tobacco price was raised by 95 cents in 1998.^29^ Further intensive tobacco price increases will be required in Japan to promote continued smoking cessation.

Previous studies found that tobacco price increases promoted smoking cessation more among the poor and the young than among the affluent and the old.^1^^,^^2^^,^^4^ However, our findings in the present study did not support these associations, with increased cessation rates observed in all groups. The early years of public health interventions, such as health information campaigns, are often damaging in terms of health equity.^5^ According to the inverse equity hypothesis,^30^ affluent sections of society preferentially benefit from, or exploit, such interventions, leading to an initial increase in inequalities (the “early stage”). Deprived sections of society only begin to catch up once affluent sections of society have extracted the maximum possible benefit (the “late stage”). Having the cheapest tobacco price of all developed countries may keep Japan in the “early stage” of the tobacco price control intervention, delaying progression to the “late stage” phase of reducing health inequality. From a health inequality perspective, further tobacco price increases are necessary in Japan.

### Limitations

There are several limitations to the study. First, smoking variables were self-reported, without biomarker validation; however, the reliability of self-reported smoking behavior was generally high.^31^ Second, we could not separate the effect of other tobacco control measures from those of the tobacco price increase. However, the weight of this effect may be small, because the only other major tobacco control measures in Japan between 2007 and 2010 were the use of varenicline and the introduction of nicotine patches in 2008.^21^ Third, the estimated association may be biased by unmeasured factors, which may contribute to the heterogeneity in findings of the present study and other tobacco price research, including industry activity to reduce price for consumers, opportunities for tax avoidance, smuggling, economic inflation, and product substitution due to wide price ranges.^3^^,^^4^ However, the influence of the former four factors might be low in Japan, because the tobacco industry did not reduce the tobacco price; it is difficult to avoid tax across national borders because Japan is an island country; smuggling into Japan is rare, although smuggling from Japan is a problem^32^; and, although it is important to account for the effects of inflation when multiple years of data are employed,^4^ changes in the rate of the inflation in Japan were very small between 2007 and 2010.^33^ An increased share of low-price tobacco products was observed in Japan after 2010 according to tobacco industry reports^34^: For example, a popular cheap brand, “Echo”, increased its share of the market by 0.5% in 2011 compared with 2010. Therefore, the impact of product substitution is expected, although the magnitude may not be large. Fourth, the longitudinal approach is ideally suited to the study of change in smoking behaviors over time. However, longitudinal studies generally include fewer subjects than other methods, so a high percentage of the subjects will be lost to follow up.^13^ If those lost to follow-up differ in important respects from those who continue to be studied, the results may be compromised.^35^

### Conclusion

We found that the 2010 tobacco price increase had a significant impact on smoking cessation among both sexes in Japan, especially those who smoked a large number of cigarettes. There were no obvious differences in the relationship between tobacco price increase and smoking cessation according to socio-demographic status, such as age or household expenditure. These findings suggest that there is an urgent need for additional tobacco price increases to reduce tobacco use and smoking inequality in Japan.

## ONLINE ONLY MATERIALS

eTable 1. Number (prevalence) of current smokers at June according to basic characteristics of total subjects.

eMaterial 1. Supplemental methods, results, reference.

eMaterial 2. Abstract and main text in Japanese.
