# Supplementary material for: Tobacco Price Increase and Smoking Cessation in Japan, a Developed Country With Affordable Tobacco: A National Population-Based Observational Study
Source: J Epidemiol. 2016 Jan 5;26(1):14–21. doi: 10.2188/jea.JE20140183 (PMC4690736; doi:10.2188/jea.JE20140183)
Supplement: eTable 1. [file je-26-014-s001.pdf]

eTable 1. Number (prevalence) of current smokers at June according to basic characteristics of total subjects

Characteristics	Men 2007 n=2,639	Men 2010 n=2,598	Men p for difference[a]	Women 2007 n=2,995	Women 2010 n=2,856	Women p for difference[a]
Current smoker at June	1080 (40.9)	961 (37.0)	0.004	355 (11.9)	306 (10.7)	0.173
Other household smoker(s)						
0 smoker	733 (36.8)	639 (31.8)	0.001	147 (8.5)	130 (7.1)	0.133
1 or more	347 (53.7)	322 (54.6)	0.775	208 (16.6)	176 (17.3)	0.653
Household expenditure						
1st (lowest) tertile	335 (44.3)	293 (36.8)	0.003	115 (14.5)	98 (11.8)	0.097
2nd tertile	338 (42.3)	316 (38.1)	0.085	104 (10.8)	99 (11.0)	0.941
3rd (highest) tertile	338 (36.7)	307 (35.7)	0.719	108 (9.7)	94 (9.3)	0.812
Missing	69 (38.8)	45 (38.8)	1.000	28 (14.6)	15 (10.9)	0.408
Age group						
20-39	378 (53.8)	317 (44.0)	0.000	149 (16.9)	122 (15.9)	0.594
40-59	459 (45.7)	397 (42.7)	0.184	151 (13.3)	130 (12.6)	0.654
60-79	243 (26.1)	247 (26.1)	1.000	55 (5.6)	54 (5.1)	0.624
Home owner						
No	226 (48.5)	226 (45.0)	0.302	118 (21.8)	97 (17.7)	0.094
Yes	854 (39.3)	735 (35.1)	0.004	237 (9.7)	209 (9.1)	0.486
Employment status						
Working	926 (44.7)	811 (40.9)	0.017	239 (14.5)	197 (12.9)	0.197
Not working	142 (27.0)	136 (24.2)	0.330	113 (8.5)	107 (8.2)	0.779
Missing	12 (30.0)	14 (25.0)	0.645	3 (12.5)	2 (6.7)	0.646
Marital status						
Married	834 (39.8)	710 (36.4)	0.030	226 (10.4)	198 (9.6)	0.442
Never married	191 (45.3)	201 (38.7)	0.046	69 (15.9)	57 (14.6)	0.629
Widowed/Divorced	55 (45.8)	50 (38.8)	0.304	60 (15.7)	51 (12.4)	0.219
Poor self-rated health						
No	914 (42.1)	826 (38.3)	0.010	281 (11.5)	254 (11.0)	0.551
Yes	129 (36.3)	97 (30.2)	0.103	62 (14.4)	44 (10.4)	0.078
Missing	37 (32.5)	38 (32.2)	1.000	12 (9.2)	8 (7.0)	0.642

[a]calculated by Fisher's exact test.
