# Supplementary material for: Tobacco Price Increase and Smoking Cessation in Japan, a Developed Country With Affordable Tobacco: A National Population-Based Observational Study
Source: J Epidemiol. 2016 Jan 5;26(1):14–21. doi: 10.2188/jea.JE20140183 (PMC4690736; doi:10.2188/jea.JE20140183)
Supplement: eMaterial 1. [file je-26-014-s002.pdf]

eMaterial 1 (online only)

Title

Tobacco price increase and smoking cessation in Japan, a developed country with affordable tobacco: a national population-based observational study

Supplementary methods

Variables

Age groups of 20-39 years-old, 40-59 years-old or 60-79 years-old were used. To assess the influence of smoking behavior of household members, the variable of living with other smoker(s) in the household was used. Number of other household smoker(s) was categorized into "0 smoker" or "1 or more smoker(s)". Equivalent household expenditure was used as a socioeconomic factor and was calculated by dividing household expenditure in May by the square root of household size, according to the method described by the Organization for Economic Cooperation and Development.[1] The study subjects were categorized into tertile according to the equivalent household expenditure. The cut-offs for the household expenditure tertile among men were 115 and 175 thousand yen per month in 2007 and 105 and 161 in 2010. Among women they were 115 and 173 in 2007 and 106 and 163 in 2010. Marital status was categorised as married, never married, or widowed or divorced. Housing tenure was categorized into "home-owner" or "not-home-owner". Employment status was divided into employed and unemployed including students and housewives. Self-rated health was surveyed as follows: "What is your current health status: excellent, very good, good, fair, or poor?" We categorized "fair" and "poor" as poor self-rated health.

Supplementary results

Number and prevalence of current smokers at June according to basic characteristics are shown in eTable 1. A statistically significant difference for the prevalence between 2007 and 2010 was observed in men in the following groups: no household smoker, lowest household expenditure tertile, 20–39 years age group, home-owner, working, married, never married, and the not-poor self-rated health group. It was not observed in women.

Supplementary References

1. Growing unequal? Income distribution and poverty in OECD countries [homepage on the Internet]. Paris: OECD; c2008 [cited 2014 30 May]. Available from: http://www.oecd.org/els/soc/growingunequalincomedistributionandpovertyinoecdcountries.htm
