# Supplementary material for: Tobacco Price Increase and Smoking Cessation in Japan, a Developed Country With Affordable Tobacco: A National Population-Based Observational Study
Source: J Epidemiol. 2016 Jan 5;26(1):14–21. doi: 10.2188/jea.JE20140183 (PMC4690736; doi:10.2188/jea.JE20140183)

eMaterial 2

http://doi.org/10.2188/jea.JE20140183　日本語版（要旨ならびに本文）

日本におけるタバコの値上げと禁煙の関連：国民生活基礎調査と国民健康栄養調査のリンケージ研究

田淵貴大[1]、中村正和[2]、中山富雄[1]、宮代勲[1]、森淳一郎[3]、津熊秀明[1]

1. 大阪府立成人病センター　がん予防情報センター
2. 地域医療振興協会 ヘルスプロモーション研究センター
3. 信州大学医学部　医学教育センター

要旨

背景：タバコ価格の禁煙に与えた影響をみた縦断研究は世界的にも少ない。本研究は、性・年齢・社会経済状況（代表的指標として等価世帯支出額 3 分位）および喫煙への依存度（代理指標として 1 日当たりの喫煙本数）に応じて、日本で 2010 年 10 月 1 日に実施されたタバコの値上げが禁煙に与えた影響を明らかにすることを目的とした。

方法：同一年の国民生活基礎調査（6 月実施）および国民健康栄養調査（11 月実施）をリンケージ（地域・世帯番号・性・年齢を使用してマッチング）した個票データを使用した。リンケージできた 2007 年および 2010 年の 6 月時点の喫煙者 2702 人が 11 月時点に禁煙したかどうか縦断的に分析した。タバコの値上げは 2010 年 10 月 1 日に実施されたため、2010 年には値上げ後の禁煙、2007 年には値上げがない状況での禁煙を評価できる。2007 年をコントロールとして、2010 年の値上げの影響について無調整および変量調整ロジスティック回帰分析によりオッズ比を計算した。

結果：喫煙者全体における禁煙実施率は 2007 年と 2010 年に大きな違いを認めた。2007 年の男性で 3.7%、女性で 9.9%であったのに対して、2010 年の男性では 10.7%、女性では 16.3%であった。喫煙への依存度のひとつの指標である 1 日当たりの喫煙本数に応じて違いが認められ、2007 年に 1 日当たり 1-10 本喫煙していた男性では 9.3%が禁煙したのに対して、11-20 本の男性では 2.7%、21 本以上の男性では 2.0%が禁煙していた。2010 年には上記と同様の喫煙本数カテゴリーに応じてそれぞれ 15.5%、10.0%、8.0%の男性が禁煙していた。変量調整ロジスティック回帰による 2010 年（2007 年＝コントロール）のオッズ比は男女ともに 1 日当たり 11 本以上吸う者で高かった。等価世帯支出額 3 分位および年齢階級別の分析においては男女で傾向が異なるなど明らかな関連を認めなかった。

結論：2010 年のタバコの値上げは男女ともに喫煙者における禁煙を顕著に増加させたものと考えられたが、社会経済状況による効果の違いは明らかではなかった。むしろヘビースモーカーにおいて高い禁煙のオッズ比が認められたことは意外であったが、これは日本のタバコが値上げ後であっても安すぎることに起因しているの

かもしれない。

背景および目的

タバコの値上げは社会経済的状況が異なる様々な人の喫煙行動にどのような影響を与えたのか？タバコの値上げの喫煙格差へのインパクトをみることは健康政策研究の重要な課題である[1,2]。タバコに対する増税（一般にタバコの値上げを伴う）は一般住民におけるタバコの消費量および喫煙格差を軽減させる最も重要なタバコ対策である[3,4]。ポピュレーションアプローチの観点から[5]、タバコの値上げは一部の住民だけでなく、裕福な人々にも貧しい人々にも効果を及ぼすと考えられる。アメリカやスコットランド、オーストラリア等における先行研究によると、タバコの値上げは裕福な人や高齢な人よりも貧しい人や若い人の喫煙率を低下させ、喫煙格差を減少させる効果があった[4]。しかしながら、年齢層や性別、学歴といった要因からみた喫煙格差はタバコの値上げによって必ず格差の縮小が認められてきたわけではない[2,6]。一般にタバコ依存度が高いことはその後の禁煙や減煙などの喫煙変化が少ないことと関連している[7]。

日本ではこれまで 1998 年にタバコ税が導入されて以降、2003 年 7 月 1 日、2006 年 7 月 1 日、2010 年 10 月 1 日の 3 回、タバコ税（タバコ価格）が引き上げられた（消費税によるタバコ価格の値上がりを除く）。同時にタバコ会社は彼らの利益のためにタバコ価格の便乗値上げを実施している。そのため、例えばマイルドセブン 20 本の価格は、2003 年に 250 円から 270 円に（8%の増加）、2006 年に 300 円に（11%の増加）、2010 年に 410 円に（37%の増加）なった[8]。これらの値上げの影響もあり、日本における成人の喫煙率は減少してきている。男性では 2001 年の 48%から 2010 年には 33%に、女性では 2001 年の 14%から 2010 年には 10%に減少している[9]。しかし、アフォーダビリティーインデックス（タバコが手頃な価格かどうかの指標）によると、日本のタバコの値段はとても安い[10]。調査された先進国の中で、2009 年における日本のタバコ価格は最も手頃であり、たった 11.5 分間働けばタバコ 20 本を買うことができる状況であった。これは 2010 年の値上げ後であっても、16 分に増えるだけであって、おおよそ 30 分が必要なオーストラリアやカナダ、オランダ等ほかの先進国の状況にははるかに及ばない[10]。日本のタバコの値段の状況はこのように先進国の中でも特殊であるため、タバコの値上げの喫煙行動および喫煙格差に与える影響も特殊かもしれない。これまで禁煙におけるタバコ価格の影響を評価した縦断調査は少ない[4, 11, 12]。そのため、日本におけるタバコの値上げの喫煙および喫煙格差に与えた影響を評価することを本研究の目的とした。

研究方法

１．対象

同一年の国民生活基礎調査（6 月実施）および国民健康栄養調査（11 月実施）をリンケージ（地域・世帯番号・性・年齢を使用してマッチング）した個票データを使用した。国民生活基礎調査は日本全国から調査地区と世帯を無作為に 2 段階抽出して世帯員全員について毎年実施される調査（世帯票）であり、喫煙については 3 年に 1 回調査されている（健康票）。2007 年 6 月（2010 年 6 月の結果を括弧内に示す）には 2005 年の国勢調査に基づく全国 94 万地点から、

5440 地点（5510 地点）がランダムに選択され、その全世帯が調査対象となり、229,821 世帯（228,864 世帯）から有効回答が得られた[有効回答率 79.9％（79.1%）]。これらの地点のうち、300 地点が国民健康栄養調査のためにランダムに抽出され（ただし、国民生活基礎調査における所得票・貯蓄票および介護票の対象世帯は除外）、同じく抽出された全世帯に対して 2007 年 11 月（2010 年 11 月）に調査が実施された[3508 世帯；有効回答率 66.5%（3684 世帯；有効回答率 68.8%）]。どちらの調査も世帯の全世帯員を調査の対象としている。

リンケージできた 2007 年および 2010 年の 6 月時点の喫煙者 2702 人が 11 月時点に禁煙したかどうか縦断的に分析した。タバコの値上げは 2010 年 10 月 1 日に実施されたため、2010 年には値上げ後の禁煙、2007 年には値上げがない状況での禁煙を評価できる（図１）。2007 年をコントロールとして、2010 年の値上げの影響について多変量調整ロジスティック回帰分析によりオッズ比を計算した。

２．喫煙の定義

まず、6月に実施された国民生活基礎調査の結果に応じて現在喫煙者と現在非喫煙者に分ける。次に11月に実施された国民健康栄養調査の結果に応じて、禁煙を定義する。すなわち、6月時点に喫煙していたが、11月時点に喫煙していなかった場合を禁煙と定義した。

３．その他の変数の定義

喫煙行動に関連する変数として [15, 16]、(i) 年齢、 (ii) 世帯支出額、 (iii) 持ち家の有無、 (iv) 就労状況、 (v) 婚姻状況 (married, never married or widowed/divorced)、 (vi) 主観的健康観、(vii) 1 日当たりの喫煙本数 (1-10, 11-20 or >=21)、(viii) 本人以外の世帯内の喫煙者数 (0 or >=1)を用いた。1 日当たりの喫煙本数はタバコ依存の代理変数として用いた [17, 18]。

４．統計解析

偶然にも、2010年の国民生活基礎調査と国民健康栄養調査の間の期間にタバコの値上げが実施されたため、その影響を評価する機会に恵まれた。特に日本を含むアジアの国々では男女の喫煙行動が大きく異なるため[19]、男女別に分析を実施した。対象者の属性および禁煙した割合（%）を上記の変数に応じて示した。2007年と2010年を比較するため、フィッシャーの正確検定を実施した。2010年のタバコの値上げの禁煙に対するオッズ比および95%信頼区間を計算するため、単変量および多変量調整のロジスティック回帰分析を2007年および2010年の両方を含めたサンプルにて実施した。2007年の6月から11月にはタバコの値上げはなかったため（図１）、2007年の禁煙状況がコントロールとして用いられた。

タバコの値上げの禁煙に与えた影響が社会経済状況やタバコ依存度によって異なるかどうか検証するため、層別化解析を実施した。世帯支出額は代表的な社会経済要因である所得の代替変数であるため[20]、社会経済要因として世帯支出額を用いた。 分析にはSAS version 9.2 (SAS Institute, Cary, NC, USA)を用いた。

個人識別のない既存の資料やデータを用いて行う研究であるので、特に倫理的な問題はない。データ使用の枠組みは公的統計資料の二次利用であり、疫学研究指針に基づき倫理審査の除外対象にあたる。データは厚生労働省からの許可のもと利用した。

結果

6 月における現在喫煙者の特性を表 1 に示した。2007 年と 2010 年で有意な分布の違いが認められた項目は、男性における 1 日当たりの喫煙本数のみであった。そのため、この項目は分析における交絡要因の条件を満たすと考えられ、調整するべき要因として扱った。

対象者の特性に応じた約 5 ヶ月のフォローアップにおける禁煙した割合（%）を表 2 に示した。男性では、2007 年に 3.7%の喫煙者が禁煙し、2010 年には 10.7%が禁煙していた。女性では、2007 年に 9.9%、2010 年には 16.3%が禁煙していた。2007 年に 1 日当たりの喫煙本数が 1-10 本の男性では 9.3%が禁煙していたのに対して、11-20 本の男性では 2.7%、21 本以上の男性では 2.0%が禁煙していた。この数字は 2010 年には、それぞれ 15.5%、10.0%、8.0%に増加していた。2007 年と 2010 年で禁煙した割合に有意な差が認められたのは、1 日当たりの喫煙本数が 11-20 本、世帯支出額が最も高い分位、持ち家なし、婚姻ありの項目であった（男女ともに）。

層別解析によって 2007 年と比較して 2010 年の禁煙に対するオッズ比を計算した結果を表 3 に示した。男性においては 1 日当たりの喫煙本数を調整したモデルが交絡要因調整の観点から適切だと考えられた。男性では 1 日当たりの喫煙本数 1-10 本以外の全ての層で 2007 年と比較して 2010 年の禁煙に対するオッズ比は有意に高い値を呈していた。モデル間で結果は大きく変わらなかった。表に提示した以外に多くの変数で調整したモデルにおいても結果はほとんど変わらなかった（data not shown）。禁煙に対するオッズ比は女性におけるよりも男性において高かった。1 日当たり喫煙本数 11 本以上では 1-10 本と比較して禁煙に対するオッズ比が男女ともに高かった。一方、世帯支出額 3 分位および年齢階級に応じたタバコの値上げの禁煙への影響は明らかな関連の方向性を認めなかった（男女ともに）。例えば、世帯支出額 3 分位の層別解析で男性では額が低い層で高いオッズ比を呈した一方、女性では反対の傾向だった。

考察

日本における 2010 年 10 月 1 日のタバコ増税（＋便乗値上げ）は、2007 年の状況と比較して、有意に禁煙を増加させたものと考えられた。この結果は従来からのタバコの値上げが最も重要なタバココントロール政策の一つであるという考えを支持するものであった[2]。日本ではたばこ事業法のもと、日本全国におけるタバコ価格の地域間の差はなく、2010 年 10 月 1 日から遅れることなく、タバコの値上げが実施されたので[21]、日本の住民全員がタバコの値上げの影響を即座に受けたものとみなせると考えた。11 月に国民健康栄養調査が実施されたので、10 月 1 日の値上げから 1 ヶ月間しか期間がないが、いわゆる予測効果（anticipation effect）[22]というものも働い

ていると考えられた。事実、値上げ前の2010年8-9月に実施された喫煙者における禁煙の意向に関する調査[23]では、53%の喫煙者がタバコの値上げを理由として２ヶ月以内に禁煙するつもりだと回答し、72%の禁煙するつもりの喫煙者はタバコの値上げの前もしくは当日に禁煙するつもりだと回答した。こうしたことから、6月から11月までのフォローアップであっても10月1日のタバコの値上げの影響を評価することができるものと考えた。2007年から2010年の間に、禁煙治療薬の保険適応が認められたことの他にタバコ対策上の大きな変化は認められていないことから[21]、2007年の状況をコントロールとする分析方法についても有用であると判断できた。

禁煙に対するオッズ比は女性におけるよりも男性において高かった。禁煙した割合の絶対値は男性よりもむしろ女性において高かったが、タバコの値上げは女性よりも男性に対してより影響を及ぼすのかもしれない。これには下記のような理由が考えられた。(1) 男性の方が女性よりも自由に使えるお金が少ないこと[24]、(2) 女性の方が男性よりもタバコの値段に関わらず健康に対する意識が高いこと[25]、(3) 女性の方が男性よりもタバコを止めるきっかけが多いのかもしれないこと（たとえば妊娠・出産など）[26]である。

一般にタバコ依存が禁煙を阻むと考えられる[7]。しかし、タバコ依存がタバコの値上げと禁煙の関連に与える影響についてはあまり知られていない[4]。本研究ではより多くのタバコを吸っている者においてより大きなタバコ値上げの禁煙に対するオッズ比が認められた。もしかするとタバコの値上げは喫煙本数が多いほど大きく影響する（金額的には当然そうなる）のかもしれない。また、日本ではタバコ価格がかなり手頃であるため[10]、タバコの値上げに敏感に反応する喫煙者が喫煙し続けていた状況があったのかもしれない。

１．政策への反映

健康日本２１（第二次）では喫煙率の減少だけでなく、健康格差の縮小も具体的な目標として掲げられた[27]。2022年には日本における喫煙率を12%にすることを目標としている。2010年の国民健康栄養調査では2010年10月のタバコの値上げに起因すると考えられる喫煙率の大幅な減少が認められたものの、2011年の調査では男女合計の喫煙率は2010年よりも増加した[14]。禁煙できても、それを長く維持することは難しい[28]。2010年のタバコの値上げでいったん禁煙しても2011年には再度吸い始めた者が多くいたのだろう。この理由としてタバコの値上げの後でも日本のタバコ価格が十分に安すぎたことが考えられる。米国カリフォルニア州における先行研究ではタバコの値上げの禁煙に与える影響は4ヶ月しか続かなかったと報告されている[29]。さらに禁煙を促してゆくためには日本におけるさらなるタバコの値上げ、増税が必要である。

先行研究ではタバコの値上げは裕福な者や高齢の者よりもむしろ貧しい者や若年の者において禁煙を促す効果が大きいとされている[1, 2, 4]。ところが、本研究ではこの方向性を指示しなかった。健康情報の啓発活動など公衆衛生政策の介入早期のフェーズだとむしろ健康

格差が拡大する可能性が指摘されている[5]。逆進的格差変動仮説 (inverse equity hypothesis)[30]によると、早期にはむしろ恵まれた集団が公衆衛生政策からのベネフィットを得て、健康格差が拡大傾向となるが、恵まれた集団が頭打ちになった後のフェーズには恵まれない集団も追い付いてきて健康格差の縮小傾向が始まる。先進国で最もタバコ価格が手ごろな状況にある日本は、上記仮説における早期の段階にあるものと考えられる。日本において健康格差を縮小させるためにも、タバコ価格の引き上げが必要であると考えられた。

２．研究の限界

第一に、本研究は自己申告による受動喫煙曝露に関する調査項目を用いたため、誤分類のリスクは否定できない。ただし、先行研究において自己申告による曝露とバイオマーカーで測定した曝露の間で高い相関が認められている[31]。第二に、本研究では考慮できていないタバコ対策の側面がある。(1) タバコ会社による消費者における実際の値段を下げる活動、(2) タバコの増税を回避する行動、(3) タバコの密輸、(4) インフレの影響、(5) タバコ価格の範囲が広いことに由来する使用するタバコ製品の変更[3,4]が該当する。しかし、日本においては初めの4つの要因の影響は小さいかもしれない。この理由として、(1) タバコ会社はタバコに景品を付けるなどキャンペーンを実施したが、値下げは実施しなかったこと、(2) 日本は島国であり、国境を越えて増税を回避する行動は困難であること、(3) 日本からの密輸は問題である[32]一方、タバコ価格の安い日本への密輸はまれであること、(4) インフレの影響を考慮することは重要である[4]が、日本における2007年と2010年の間のインフレ率はほとんど変化がなかった[33]こと、が挙げられる。5番目の観点に関して、2010年の値上げ以降、低価格帯のタバコブランド製品の消費（シェア）が増えていることが報告された[34]。例えば、2010年と比較して2011年には代表的な低価格ブランド「エコー」のシェアは0.5%増加した。これらの影響は考えられるものの大きな影響があるとは考えにくい。第三に、継時的な喫煙状況の変化（すなわち禁煙）を評価するのに縦断調査デザインは適している。しかし、一般に縦断調査はサンプル数が少なく、追跡不能となる割合も高いことが多い[13]。このことで結果にバイアスがあることは否定できない[35]。

結論

日本における2010年のタバコの値上げは男女両方において禁煙するかどうかに有意な影響を及ぼしていたと分かった。特に1日当たりの喫煙本数が多い者でのインパクトが大きかった。一方、タバコ値上げの禁煙に与えた影響は、世帯支出額や年齢階級に応じて大きな違いがあるかどうかは明らかではなかった。これらの結果は日本における喫煙率および喫煙格差を減らすために、さらなるタバコの値上げが必要であることを示しているものと考えられる。なぜなら、これらの結果は日本のタバコ価格が安すぎることに起因しているとも考えられるからである。

引用文献

1. Hill S, Amos A, Clifford D, Platt S. Impact of tobacco control interventions on socioeconomic inequalities in smoking: review of the evidence. Tob Control. 2014;23(e2):e89-97.

2. Thomas S, Fayter D, Misso K, Ogilvie D, Petticrew M, Sowden A, et al. Population tobacco control interventions and their effects on social inequalities in smoking: systematic review. Tob Control. 2008;17:230-7.

3. Wilson LM, Avila Tang E, Chander G, Hutton HE, Odelola OA, Elf JL, et al. Impact of tobacco control interventions on smoking initiation, cessation, and prevalence: a systematic review. J Environ Public Health. 2012;2012:961724.

4. International Agency for Research on Cancer. IARC Handbooks of Cancer Prevention Tobacco Control Volume 14: Effectiveness of Tax and Price Policies for Tobacco Control. Lyon, France 2011.

5. Frohlich KL, Potvin L. Transcending the known in public health practice: the inequality paradox: the population approach and vulnerable populations. Am J Public Health. 2008;98:216-21.

6. Amos A, Greaves L, Nichter M, Bloch M. Women and tobacco: a call for including gender in tobacco control research, policy and practice. Tob Control. 2012;21:236-43.

7. Vangeli E, Stapleton J, Smit ES, Borland R, West R. Predictors of attempts to stop smoking and their success in adult general population samples: a systematic review. Addiction. 2011;106:2110-21.

8. Ito Y, Nakamura M. The effect of increasing tobacco tax on tobacco sales in Japan. Nihon Koshu Eisei Zasshi. 2013;60:613-8 (in Japanese).

9. Ministry of Health, Labour and Welfare. Comprehensive survey of living condition of people on health and welfare. Tokyo 2014 [cited 2014 May 9]. Available from: http://www.mhlw.go.jp/toukei/list/20-21.html.

10. Eriksen M, Mackay J, Ross H. The Tobacco Atlas, Fourth Edition. Atlanta, USA: American Cancer Society; 2012.

11. Ross H, Blecher E, Yan L, Hyland A. Do cigarette prices motivate smokers to quit? New evidence from the ITC survey. Addiction. 2011;106:609-19.

12. Dunlop SM, Perez D, Cotter T. Australian smokers' and recent quitters' responses to the increasing price of cigarettes in the context of a tobacco tax increase. Addiction. 2011;106:1687-95.

13. Nishi N, Nakade M, Sarukura N, Nozue M, Tsubota M, Miyoshi M, et al. Response rate and the related factors of National Health and Nutritional Survey. Kosei no Shihyo. 2012;59:10-5 (in Japanese).

14. Ministry of Health, Labour and Welfare. National Health and Nutritional Survey Tokyo 2014 [cited 2014 May 9]. Available from: http://www.mhlw.go.jp/bunya/kenkou/kenkou_eiyou_chousa.html.

15. Fukuda Y, Nakamura K, Takano T. Socioeconomic pattern of smoking in Japan: income inequality and gender and age differences. Ann Epidemiol. 2005;15:365-72.

16. Fukuda Y, Nakao H, Imai H. Different income information as an indicator for health inequality among Japanese adults. J Epidemiol. 2007;17:93-9.

17. Durkin SJ, Biener L, Wakefield MA. Effects of different types of antismoking ads on reducing disparities in smoking cessation among socioeconomic subgroups. Am J Public Health. 2009;99:2217-23.

18. Heatherton TF, Kozlowski LT, Frecker RC, Fagerström KO. The Fagerstrom Test for Nicotine Dependence: a revision of the Fagerstrom Tolerance Questionnaire. Br J Addict. 1991;86:1119-27.

19. International Agency for Research on Cancer. A

review of human carcinogens. Part E: Personal habits and indoor combustions. Lyon, France 2012.
20. Fukuda Y, Hiyoshi A. Associations of household expenditure and marital status with cardiovascular risk factors in Japanese adults: analysis of nationally representative surveys. J Epidemiol. 2013;23:21-7.
21. Katanoda K, Jiang Y, Park S, Lim MK, Qiao YL, Inoue M. Tobacco control challenges in East Asia: proposals for change in the world's largest epidemic region. Tob Control. 2014;23:359-68.
22. Abadie A, Diamond A, Hainmueller J. Synthetic Control Methods for Comparative Case Studies: Estimating the Effect of California's Tobacco Control Program. J Am Stat Assoc. 2010;105:493-505.
23. Pfizer Japan Inc. Internet survey for Japanese smokers before tobacco tax increase in 2010 2010 [cited 2014 16 September]. Available from: http://www.pfizer.co.jp/pfizer/company/press/2010/2010_09_16.html.
24. Softbrain field co. ltd. Spending money in husband and wife Tokyo2012 [cited 2013 10 December]. Available from: http://www.sbfield.co.jp/news/2012/06/29_130000.html.
25. Eguchi E, Iso H, Tanabe N, Wada Y, Yatsuya H, Kikuchi S, et al. Healthy lifestyle behaviours and cardiovascular mortality among Japanese men and women: the Japan collaborative cohort study. Eur Heart J. 2012;33:467-77.
26. Kaneko A, Kaneita Y, Yokoyama E, Miyake T, Harano S, Suzuki K, et al. Smoking trends before, during, and after pregnancy among women and their spouses. Pediatr Int. 2008;50:367-75.
27. Ministry of Health, Labour and Welfare. Health Japan 21 (Second) 2012 [cited 2012 6 Aug]. Available from: http://www.mhlw.go.jp/bunya/kenkou/kenkounippon21.html.
28. Stapleton J. Cigarette smoking prevalence, cessation and relapse. Stat Methods Med Res. 1998;7:187-203.
29. Reed MB, Anderson CM, Vaughn JW, Burns DM. The effect of cigarette price increases on smoking cessation in California. Prev Sci. 2008;9:47-54.
30. Victora CG, Vaughan JP, Barros FC, Silva AC, Tomasi E. Explaining trends in inequities: evidence from Brazilian child health studies. Lancet. 2000;356:1093-8.
31. Caraballo RS, Giovino GA, Pechacek TF, Mowery PD. Factors associated with discrepancies between self-reports on cigarette smoking and measured serum cotinine levels among persons aged 17 years or older: Third National Health and Nutrition Examination Survey, 1988-1994. Am J Epidemiol. 2001;153:807-14.
32. Wen CP, Peterson RA, Cheng TY, Tsai SP, Eriksen MP, Chen T. Paradoxical increase in cigarette smuggling after the market opening in Taiwan. Tob Control. 2006;15:160-5.
33. OECD. OECD Data Lab Paris2013 [cited 2013 15 Nov]. Available from: http://www.oecd.org/statistics/datalab/.
34. Tobacco Institute of Japan. Cigarette statistics in Japan Tokyo2013 [cited 2013 15 Nov]. Available from: http://www.tioj.or.jp/data/index.html.
35. International Agency for Research on Cancer. IARC Handbooks of Cancer Prevention Tobacco Control Volume 13: Evaluating the Effectiveness of Smoke-free Policies. Lyon, France 2009.

表 1. 6 月における現在喫煙者の特性：対象者数（%）

特性	男性 2007 n=1080	男性 2010 n=961	男性 p for difference[a]	女性 2007 n=355	女性 2010 n=306	女性 p for difference[a]
1 日当たりの喫煙本数			0.009			0.257
1-10 本	205 (19.0)	219 (22.8)		121 (34.1)	125 (40.9)	
11-20 本	523 (48.4)	490 (51.0)		186 (52.4)	150 (49.0)	
21 本以上	348 (32.2)	249 (25.9)		45 (12.7)	29 (9.5)	
Missing	4 (0.4)	3 (0.3)		3 (0.9)	2 (0.7)	
本人以外の世帯内喫煙者数			0.509			0.813
0 人	733 (67.9)	639 (66.5)		147 (41.4)	130 (42.5)	
1 人以上	347 (32.1)	322 (33.5)		208 (58.6)	176 (57.5)	
世帯支出額 3 分位			0.369			0.434
1st (lowest) 3 分位	335 (31.0)	293 (30.5)		115 (32.4)	98 (32.0)	
2nd 3 分位	338 (31.3)	316 (32.9)		104 (29.3)	99 (32.4)	
3rd (highest) 3 分位	338 (31.3)	307 (32.0)		108 (30.4)	94 (30.7)	
Missing	69 (6.4)	45 (4.7)		28 (7.9)	15 (4.9)	
年齢階級			0.230			0.727
20-39	378 (35.0)	317 (33.0)		149 (42.0)	122 (39.9)	
40-59	459 (42.5)	397 (41.3)		151 (42.5)	130 (42.5)	
60-79	243 (22.5)	247 (25.7)		55 (15.5)	54 (17.7)	
持ち家			0.165			0.678
No	226 (20.9)	226 (23.5)		118 (33.2)	97 (31.7)	
Yes	854 (79.1)	735 (76.5)		237 (66.8)	209 (68.3)	
就労状況			0.621			0.662
働いている	926 (85.7)	811 (84.4)		239 (67.3)	197 (64.4)	
働いていない	142 (13.2)	136 (14.2)		113 (31.8)	107 (35.0)	
Missing	12 (1.1)	14 (1.5)		3 (0.9)	2 (0.7)	
婚姻状況			0.174			0.967
結婚している	834 (77.2)	710 (73.9)		226 (63.7)	198 (64.7)	
未婚	191 (17.7)	201 (20.9)		69 (19.4)	57 (18.6)	
離婚･死別	55 (5.1)	50 (5.2)		60 (16.9)	51 (16.7)	
主観的健康観｢悪い｣			0.354			0.466
No	914 (84.6)	826 (86.0)		281 (79.2)	254 (83.0)	
Yes	129 (11.9)	97 (10.1)		62 (17.5)	44 (14.4)	
Missing	37 (3.4)	38 (4.0)		12 (3.4)	8 (2.6)	

[a]p for difference は Fisher の正確検定による.

表 2. 対象者の特性に応じた約 5 ヶ月のフォローアップにおける禁煙した割合（%）

Characteristics	喫煙者における禁煙した割合 (%)							
	男性				女性			
	2007 %	2010 %	増加 % point	p for difference[a]	2007 %	2010 %	増加 % point	p for difference[a]
全体	3.7	10.7	7.0	<0.001	9.9	16.3	6.5	0.014
1 日当たりの喫煙本数								
1-10 本	9.3	15.5	6.3	0.057	18.2	22.4	4.2	0.432
11-20 本	2.7	10.0	7.3	<0.001	5.9	13.3	7.4	0.023
21 本以上	2.0	8.0	6.0	0.001	2.2	3.5	1.2	-
本人以外の世帯内喫煙者数								
0 人	4.1	11.3	7.2	<0.001	12.2	18.5	6.2	0.180
1 人以上	2.9	9.6	6.8	0.000	8.2	14.8	6.6	0.051
世帯支出額 3 分位								
1st (lowest) 3 分位	3.0	12.0	9.0	<0.001	11.3	12.2	0.9	0.835
2nd tertile	3.0	8.5	5.6	0.002	5.8	13.1	7.4	0.092
3rd (highest) 3 分位	4.7	12.4	7.7	0.001	10.2	24.5	14.3	0.008
年齢階級								
20-39	3.7	10.1	6.4	0.001	9.4	16.4	7.0	0.098
40-59	2.8	9.8	7.0	<0.001	8.6	13.9	5.2	0.184
60-79	5.4	13.0	7.6	0.005	14.6	22.2	7.7	0.332
持ち家								
No	2.7	9.7	7.1	0.003	5.9	14.4	8.5	0.041
Yes	4.0	11.0	7.0	<0.001	11.8	17.2	5.4	0.107
就労状況								
働いている	3.1	10.7	7.6	<0.001	8.4	13.2	4.8	0.118
働いていない	7.0	11.0	4.0	0.297	13.3	22.4	9.2	0.081
婚姻状況								
結婚している	3.1	11.6	8.4	<0.001	11.1	19.7	8.6	0.015
未婚	5.8	8.5	2.7	0.332	8.7	14.0	5.3	0.401
離婚・死別	5.5	8.0	2.6	0.706	6.7	5.9	-0.8	-
主観的健康観「悪い」								
No	3.7	10.4	6.7	<0.001	11.0	16.9	5.9	0.080
Yes	3.1	9.3	6.2	0.060	4.8	13.6	8.8	0.158

[a]p for difference は Fisher の正確検定による.

表 3. 層別解析による 2007 年と比較した 2010 年の禁煙に対するオッズ比（95%信頼区間）

層別化変数	男性	女性
	2010 (ref = 2007)	2010 (ref = 2007)
合計	n=2034	n=656
無調整	3.12 (2.14, 4.55)	1.80 (1.13, 2.87)[a]
1 日当たりの喫煙本数を調整	3.01 (2.06, 4.39)[a]	1.68 (1.04, 2.70)
さらに年齢を調整	3.09 (2.12, 4.50)	1.78 (1.12, 2.85)
1 日当たりの喫煙本数 ***1-10 本***	n=424	n=246
無調整	1.80 (0.99, 3.27)[a]	1.30 (0.70, 2.43)[a]
年齢を調整	1.84 (1.01, 3.35)	1.29 (0.69, 2.41)
1 日当たりの喫煙本数 ***11-20 本***	n=1013	n=336
無調整	4.04 (2.20, 7.42)[a]	2.45 (1.13, 5.29)[a]
年齢を調整	3.93 (2.14, 7.23)	2.40 (1.11, 5.19)
1 日当たりの喫煙本数 ***21 本以上***	n=597	n=74
無調整	4.26 (1.77, 10.23)[a]	1.57 (0.09, 26.15)[a]
年齢を調整	4.15 (1.72, 10.00)	NC
世帯支出額 3 分位, lowest	n=628	n=212
無調整	4.41 (2.14, 9.07)	1.00 (0.43, 2.35)[a]
1 日当たりの喫煙本数を調整	4.52 (2.19, 9.35)[a]	NC
さらに年齢を調整	4.50 (2.18, 9.31)	NC
世帯支出額 3 分位, middle	n=650	n=201
無調整	3.07 (1.46, 6.45)	2.47 (0.90, 6.79)[a]
1 日当たりの喫煙本数を調整	2.92 (1.38, 6.17)[a]	2.82 (1.00, 7.94)
さらに年齢を調整	2.94 (1.39, 6.22)	2.84 (1.01, 8.03)
世帯支出額 3 分位, highest	n=642	n=201
無調整	2.84 (1.55, 5.20)	2.83 (1.29, 6.18)[a]
1 日当たりの喫煙本数を調整	2.69 (1.45, 4.99)[a]	NC
さらに年齢を調整	2.67 (1.43, 4.97)	NC
年齢階級, 20-39 歳	n=691	n=267
無調整	2.94 (1.54, 5.62)	1.94 (0.92, 4.11)[a]
1 日当たりの喫煙本数を調整	2.64 (1.37, 5.08)[a]	NC
年齢階級, 40-59 歳	n=856	n=280
無調整	3.74 (1.97, 7.11)	1.69 (0.80, 3.61)[a]
1 日当たりの喫煙本数を調整	3.55 (1.86, 6.76)[a]	NC
年齢階級, 60-79 歳	n=487	n=109
無調整	2.60 (1.33, 5.08)	1.68 (0.63, 4.50)[a]
1 日当たりの喫煙本数を調整	2.77 (1.41, 5.47)[a]	1.65 (0.60, 4.51)

略語：NC，収束せず.

図１．本研究に関する時間の流れ

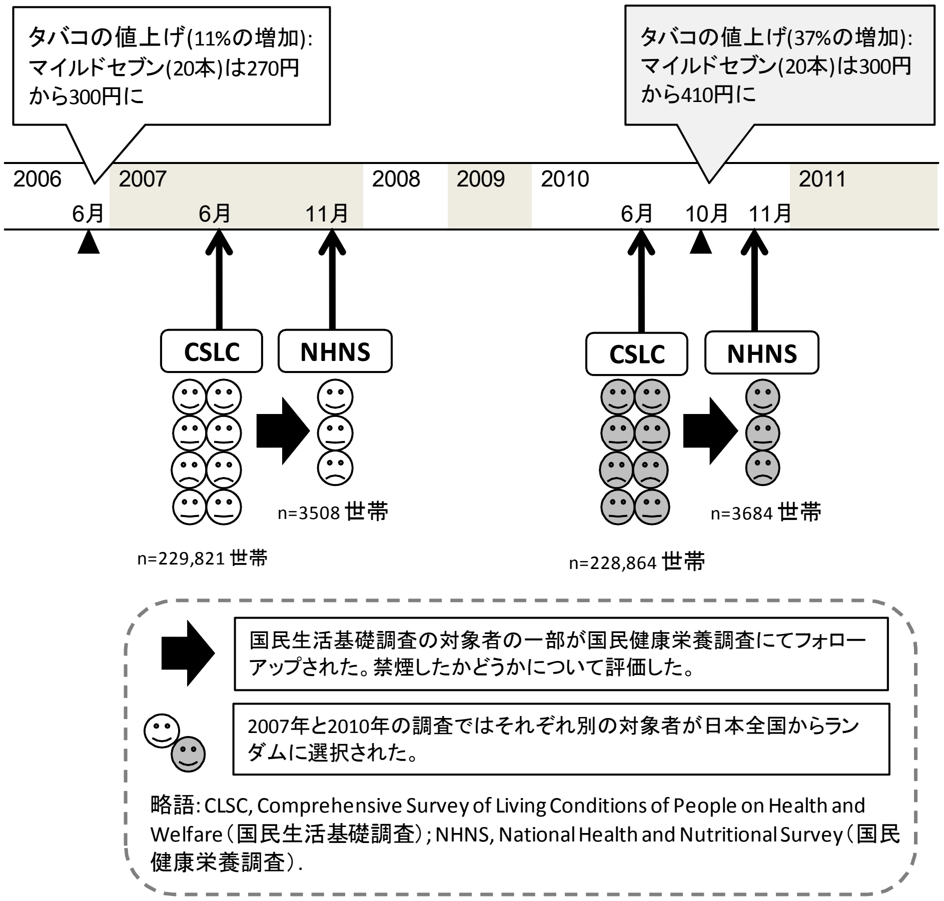

Supplement: eMaterial 2. [file je-26-014-s003.pdf]
